# Learning changes the attentional status of prospective memories

**DOI:** 10.3758/s13423-016-1008-7

**Published:** 2016-02-18

**Authors:** Dirk van Moorselaar, Jan Theeuwes, Christian N. L. Olivers

**Affiliations:** Department of Experimental and Applied Psychology, VU University, Van der Boechorststraat 1, 1081 BT Amsterdam, The Netherlands

**Keywords:** Visual working memory, Visual attention, Visual search

## Abstract

Objects in visual working memory (VWM) that are only prospectively relevant can nevertheless affect the guidance of attention in an ongoing visual search task. Here we investigated whether learning changes the attentional status of such prospective memories. Observers performed a visual search while holding an item in memory for a later memory test. This prospective memory was then repeated for several trials. When the memory was new, it interfered with the ongoing search task. However, with repetition, memory performance increased but memory-based interference rapidly diminished, suggesting that observers learned to shield the prospective memory from the ongoing task. This contrasts with earlier findings showing stronger attentional biases from learned memories when these are immediately task-relevant. Interestingly, interference resurfaced again in anticipation of a new memory, suggesting a reactivation of VWM. These effects were sensitive to task context, indicating that the attentional status of prospective memories is flexible.

**Electronic supplementary material:**

The online version of this article (doi:10.3758/s13423-016-1008-7) contains supplementary material, which is available to authorized users.

Visual working memory (VWM) is assumed to play an important role in guiding selective attention toward task-relevant visual objects—for example, in visual search (Bundesen, Habekost, & Kyllingsbæk, 2005; Desimone & Duncan, 1995; Wolfe, 1994; Woodman & Chun, 2006). However, VWM is not the only memory system guiding attention, since long-term memory (LTM) can also bias selection during visual search (Hutchinson & Turk-Browne, 2012; Olivers, 2011).

Learning through repetition induces a transition from VWM to LTM (Shiffrin & Schneider, 1977). Recent electroencephalography studies have indicated that this transition is remarkably rapid, since the contralateral delay activity (CDA, a marker of VWM) drops to asymptote within a handful of trials (Carlisle, Arita, Pardo, & Woodman, 2011; Gunseli, Meeter, & Olivers, 2014; Gunseli, Olivers, & Meeter, 2014; Reinhart & Woodman, 2014). Moreover, this reduction in VWM-related activity appears to be accompanied by a reduction in the P170, which has been interpreted as a marker of a more implicit longer-term memory (Woodman, Carlisle, & Reinhart, 2013; though see Gunseli, Olivers, & Meeter, 2014). In line with this, target repetitions have led to robust, automatic, and largely implicit selection benefits in visual search (Kruijne & Meeter, 2015; Maljkovic & Nakayama, 1994; Müller, Krummenacher, & Heller, 2004; Theeuwes, 2013).

So far, studies of attentional learning have investigated conditions in which the memory representation is directly relevant for the search—that is, the *target* is what is learned. However, for the cognitive system to be adaptive, it must be able to distinguish currently relevant learned information from currently irrelevant learned information. Here we investigated the influence on attention from memories that are being learned for a *prospective* task, but that are not relevant for the current task. Specifically, how does learning an item for such a prospective task affect attentional priorities?

To answer this question, we made use of an established paradigm in which observers search for a specific target, while holding an *accessory* item in memory for a later test. Thus, the accessory memory item is only relevant in a prospective sense. Nevertheless, attention during search has been shown to be inadvertently biased toward distractors that match the accessory memory item, resulting in interference (Olivers, Meijer, & Theeuwes, 2006; Soto, Heinke, Humphreys, & Blanco, 2005; but see Downing & Dodds, 2004; Woodman & Luck, 2007). The evidence suggests that the prospective relevance of the accessory memory causes it to be kept active in VWM, from where it guides attention. However, it is unknown how learning changes the attentional status of such prospective memories. There seem to be two possibilities: (1) Learning strengthens the prospective memory but is blind to task relevance, and thus results in *increased* interference stemming from prospective memories. (2) Learning strengthens the prospective memory, but at the same time observers also learn to shield it from currently ongoing tasks. In this case, as the prospective memory is deactivated within VWM, it no longer drives visual attention, and interference should *decrease*.

Figure 1 illustrates the basic procedure. Each trial began by presenting a colored item (the accessory memory), which had to be recalled at the end of the trial. In between, participants switched tasks and searched for a diamond-shaped target among disk-shaped distractors. One distractor carried a color that could match the accessory memory. Crucially, the accessory memory item was then repeated for eight trials. This led to learning, resulting in better memory performance. In Experiment 1, we tested whether learning the accessory item led to *more* interference (consistent with a relevance-blind learning mechanism) or *less* interference (consistent with a learned shielding mechanism) with the current task. In Experiments 2 and 3, we then tested the extent to which the attentional guidance from learned accessory items is under cognitive control.

**Fig. 1:**
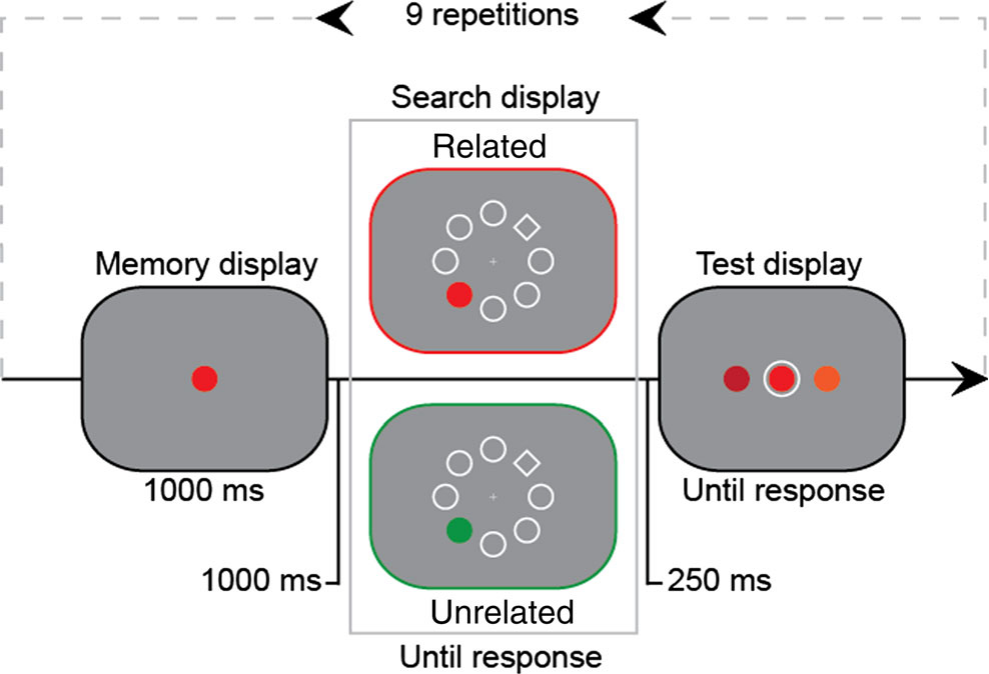
Sequence of events in a trial of Experiment 1. Both distractor type conditions are displayed

## Experiments 1A and 1B: reduced interference from learned memories

### Method

#### Participants

A planned number of 15 volunteers (14 females, one male; age 18–26 years, *M* = 21 years) participated in Experiment 1A, and another 15 volunteers (13 females, two males; age 18–24 years, *M* = 21 years) participated in Experiment 1B, in exchange for course credit or €8 per hour. The participants reported normal or corrected-to-normal acuity. Procedures were approved by the Scientific and Ethical Review Committee (Faculty of Behavioral and Movement Sciences, VU University).

#### Apparatus, stimuli, procedure, and design

A Windows 7 PC running OpenSesame v0.28 (Mathôt, Schreij, & Theeuwes, 2012) generated the stimuli on a Samsung SyncMaster 2233 120-Hz screen, at 70 cm viewing distance. Participants sat in a dimly lit cubicle. The screen background was gray (29 cd/m^2^).

A trial started with a 500-ms black fixation dot with a white (99 cd/m^2^) rim (0.11° × 0.11°). Then a memory item was presented for 1,000 ms at the center. After a 1,000-ms delay, a search display was shown, until response. The fixation dot then reappeared for 250 ms, or, in the case of an error, the word “incorrect” (in red). Finally, a memory test was shown until response.

The memory item was a colored disk (radius 1.16°) and remained identical for nine trials (Repetition factor). At the start of a sequence, a color was selected at random from five color categories (red, green, yellow, blue, and purple), with the restriction that the selected category differed from the preceding one. Within each category, the specific hue and chroma varied randomly between any of nine different combinations, chosen on the basis of the Munsell color system (Munsell Color, 1929), such that the brightness of each color was kept constant at around 26 cd/m^2^, except for yellow (66 cd/m^2^).

The search display consisted of seven distractor disks (radius 1.16°) and one diamond-shaped target (2.91° × 2.91°), all white-rimmed and all placed on an imaginary circle (radius 4.65°) centered on fixation (Theeuwes, 1992). The target was placed at one of four possible locations (i.e., top left, bottom left, top right, and bottom right). Participants were instructed to localize the diamond as quickly and accurately as possible. The target locations were coupled to keys on the QWERTY keyboard (Q for top left, A for bottom left, P for bottom right, and L for bottom left). One distractor disk was colored. The position of the colored distractor was random but at least two positions away from the target. Crucially, there were two *distractor type* conditions. In the *related* condition, the distractor color was the to-be-memorized one. In the *unrelated* condition, the color was chosen from a different category.

To limit the number of cells, only the first, third, fifth, seventh, and ninth trials in the repetition sequence could contain a memory-matching distractor. In Experiment 1A, the unrelated color was selected randomly on each trial. In Experiment 1B, the unrelated color was fixed for the odd-numbered trials, to make sure that both distractor colors (i.e., related and unrelated) were presented equally often, and thus that the relevant effects in our paradigm were not due to the fact that one type of distractor simply occurred more often (Vatterott & Vecera, 2012).

Each trial ended with a forced choice recognition task in which participants had to select the memory-matching color from three colored disks. Participants could select the memory-matching exemplar by moving an outline by pressing either A (to the left) or L (to the right; random starting point), and could submit their response with either Q or P. Participants thus could keep their fingers on the relevant buttons throughout the trial.

In both experiments, participants completed 18 practice trials and ten experimental blocks of 54 trials each. Each experimental block contained six memory displays: three related and three unrelated distractor trials per repetition (randomly mixed). This resulted in 30 trials per repetition for each distractor condition. After each block, feedback was given on reaction times (RTs; search) and accuracy (search and memory). Participants were encouraged to take a break between blocks.

### Results and discussion

**Fig. 2:**
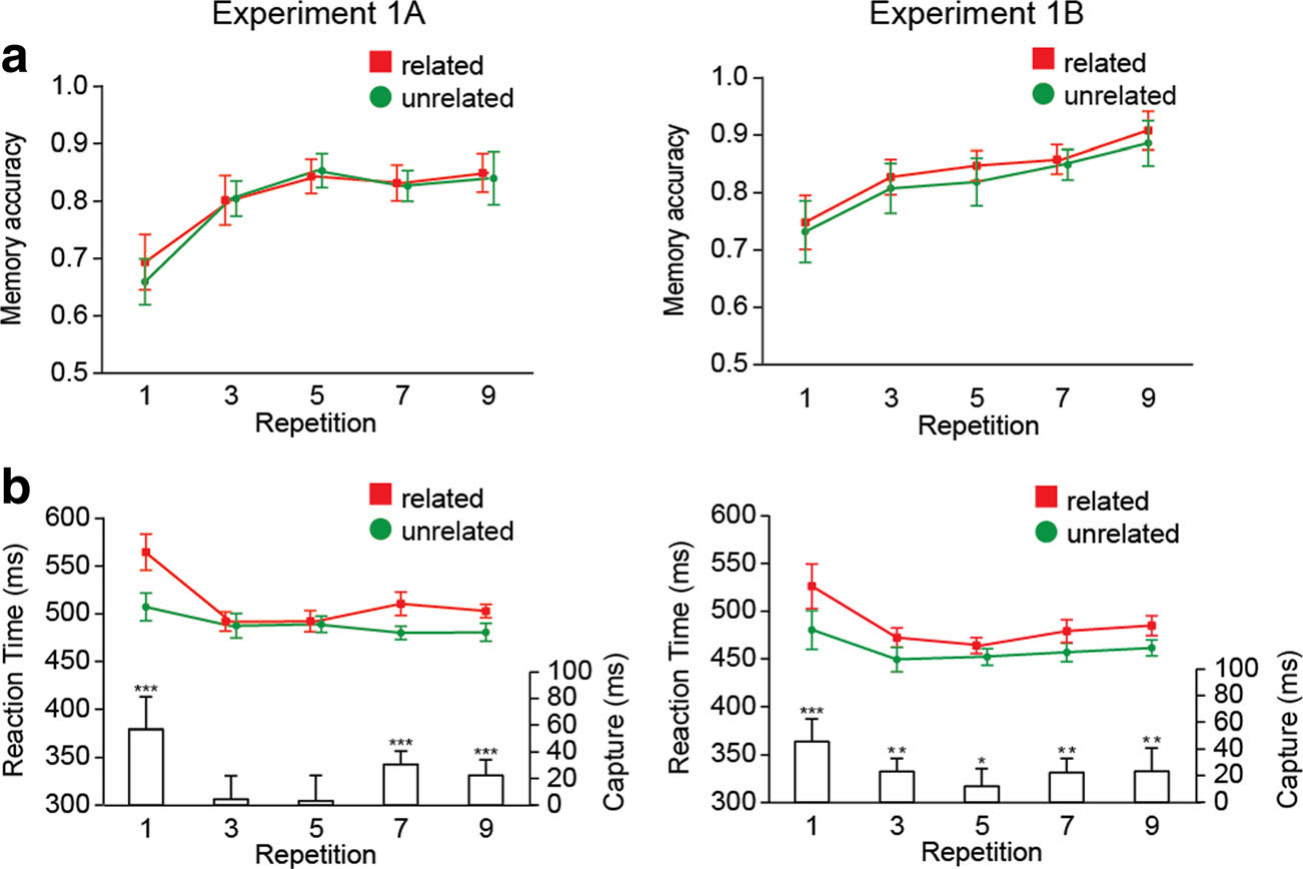
Experiments 1A and 1B: (**a**) Memory accuracy and (**b**) reaction times (RTs) as a function of repetition and distractor type in Experiments 1A (left) and 1B (right). Bars show the amounts of capture, as indexed by the difference between related and unrelated RTs. Error bars in all figures represent condition-specific, within-subjects 95 % confidence intervals (Morey, 2008). ^*^ *p* < .05, ^**^ *p* < .01, ^***^ *p* < .001. The same descriptions apply to the figures for Experiments 2 and 3

Figure 2a shows how memory performance improved with repetition. Analyses of memory and search accuracy are reported in the supplementary material. Here we focus on mean RTs in the search task.

#### Search RTs

Only odd-numbered trials in the sequence of nine trials were analyzed, since only those could contain related distractors. Incorrect trials were removed (Exp. 1A = 1.5 %, Exp. 1B = 1.3 %), and then a two-step trimming procedure was applied. First, trials with RTs shorter than 200 ms and longer than 5,000 ms were excluded. Next, the data were trimmed on the basis of a cutoff value of 2.5 standard deviations (*SD*s) from the mean RT per participant per condition (Exp. 1A = 1.3 %, Exp. 1B = 2.9 %), resulting in an overall loss of 3.5 % of trials.

The remaining RTs were entered into a repeated measures analysis of variance (ANOVA) with the factors Distractor Type (related, unrelated) and Repetition (1, 3, 5, 7, 9), and with *α* = .05. A Greenhouse–Geisser correction was applied in the case of sphericity violations. Experiment (1A, 1B) was added as a between-subjects factor to assess whether any effects were due to repetition of the distractor in the search display, rather than in memory. We observed no main effect of experiment (*F* = 1.44, *p* = *.*24), nor did it interact with any of the other factors (all *F*s < 1.67, all *p*s > .16), indicating that there was no effect of distractor repetition per se.

We did find a main effect of distractor type [*F*(1, 28) = 69.39, *p* < .001, *η*_p_^2^ = .71], reflecting overall slower RTs in the related relative to the unrelated distractor condition. The main effect of repetition was also significant [*F*(4, 112) = 27.95, *p* < .001, *η*_p_^2^ = .50], as RTs were modulated by repetition of the accessory memory item. Importantly, the distractor type by repetition interaction was highly significant [*F*(4, 112) = 12.30, *p* < .001, *η*_p_^2^ = .31]. This pattern also held when the experiments were analyzed separately (all *p*s < .01; see the supplementary material).

As can be seen from Fig. 2b, memory-based interference with search decreased with repetition. It thus appears that transferring an accessory memory from VWM to LTM makes it easier to ignore memory-matching distractors. Also, consistent with previous studies (e.g., Carlisle et al., 2011), this transition was rather rapid, occurring within one or two trials.

However, Fig. 2b also suggests that as learning progressed, interference did not continue to decrease, but reemerged somewhat toward the end of the sequence. An ANOVA in which we only entered Repetitions 5, 7, and 9 indeed suggested an interaction [*F*(2, 56) = 6.10, *p* < .01], but we emphasize that this analysis was post hoc. This resurgence was unexpected, and we will return to it after Experiment 2. However, here it could have driven the distractor type by repetition interaction, so to make sure that the initial repetition-driven decline in interference was reliable, we repeated the ANOVA, but only for Repetitions 1 to 5. This again revealed a significant distractor type by repetition interaction [*F*(2, 56) = 18.06, *p* < .001].

## Experiment 2: interference from learned prospective memories is under cognitive control

Experiment 1 showed that, with repetition, a prospective memory ceases to interfere with the current task. In Experiment 2, we tested the extent to which learning-based decrement in interference is under cognitive control. With a relatively long series of repetitions, as in Experiment 1, offloading the accessory memory to LTM as soon as possible may be a viable strategy. However, we hypothesized that when memory content is more changeable, observers may choose to keep VWM online, to anticipate updating with the new content and prevent spurious learning of soon-to-be-irrelevant representations (Braver & Cohen, 2000; O’Reilly & Frank, 2006). An adaptive system should thus be able to choose between VWM- and LTM-based processing, depending on the task context. To test this, in Experiment 2 we compared a blocked condition in which the accessory memory item was repeated for nine trials (as in Exp. 1) to a blocked condition in which the memory changed every three trials. On the basis of Experiment 1, we predicted a repetition-related decrease in interference in the nine-repetitions block. We were also curious whether we could replicate the resurgence of interference near the end of the sequence. However, for the three-repetitions condition, in which items changed frequently, we hypothesized that observers are inclined to keep VWM involved, and thus memory-based interference was predicted to be sustained across repetitions.

### Method

The method was the same as in Experiment 1A, except for the following changes: Eighteen new volunteers (15 females, three males; age 18–26 years, *M* = 21 years) participated. The participants completed separate blocks, in which the memory color was repeated either three or nine times. Note that the three- and the nine-repetitions blocks only differed in the number of repetitions. All participants completed 16 experimental blocks (eight blocks for each condition), in counterbalanced order. Each block contained six memory displays, such that a three-repetitions block contained 18 trials and a nine-repetitions block contained 54 trials. This resulted in 24 trials per repetition for each distractor condition.

### Results and discussion

**Fig. 3:**
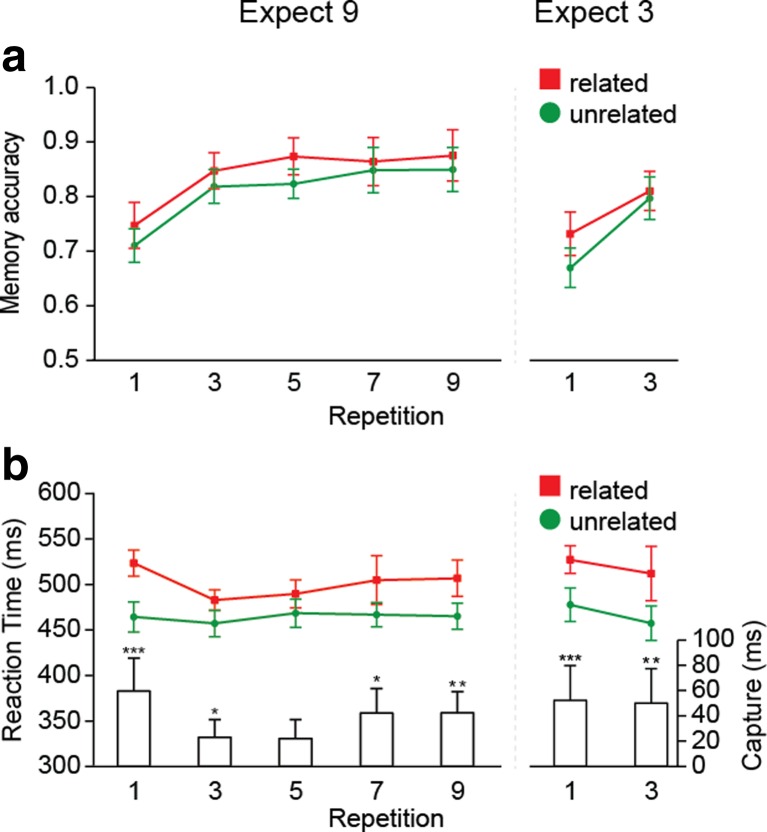
Experiment 2: (**a**) Memory accuracy and (**b**) reaction times as a function of repetition and distractor type. The results from the nine-repetitions blocks (left) are shown separately from the results in the three-repetitions blocks (right)

Figure 3 shows the RT results. Exclusion of incorrect search trials (2.0 %) and data trimming (2.6 %) resulted in an overall loss of 3.8 % of the data. In the first step of the analysis, we focused only on the first three repetitions in both sets of blocks. An ANOVA with the within-subjects factors Block Type (nine repetitions, three repetitions), Distractor Type (related, unrelated), and Repetition (1, 3) showed a main effect of distractor type [*F*(1, 17) = 20.54, *p* < .001, *η*_p_^2^ = .55] and a main effect of repetition [*F*(1, 17) = 22.05, *p* < .001, *η*_p_^2^ = .57], but no effect of block type (*F* = 1.20, *p* > *.*25). The three-way interaction was significant [*F*(1, 17) = 5.78, *p* = *.*028, *η*_p_^2^ = .25]. Splitting up the analyses revealed a distractor type by repetition interaction in the nine-repetitions blocks [*F*(1, 17) = 10.23, *p* = *.*005, *η*_p_^2^ = .38], but not in the three-repetitions blocks (*F* = 0.04, *p* > *.*25), where there were only main effects of distractor type [*F*(1, 17) = 15.86, *p* = *.*001, *η*_p_^2^ = .48] and repetition [*F*(1, 17) = 14.37, *p* = *.*001, *η*_p_^2^ = .46]. When the memory item was repeated three times, a related distractor slowed RTs to the same extents on the first and third trials of the sequence (*t* = 0.19, *p* > *.*25). In contrast, when the memory item was repeated nine times, memory-based interference was significantly reduced on the third relative to the first repetition [*t*(17) = 3.20, *p* < .01]. Analyzing the nine-repetitions blocks separately revealed main effects of distractor type [*F*(1, 17) = 11.85, *p* = *.*003, *η*_p_^2^ = .41] and repetition [*F*(4, 68) = 5.06, *p* = *.*001, *η*_p_^2^ = .30], and also a significant interaction [*F*(3, 44) = 3.64, *p* = *.*025, *η*_p_^2^ = .18].

These results show that the repetition-based decrement in interference from prospective memories depends on the anticipated length of the repetition sequence. Consistent with our hypothesis, when the memory task changed frequently, memory-based interference did not diminish, whereas it did for more stable sequences of nine representations. This suggests that, with learning, a prospective memory is not automatically shielded from the current task, but that this transfer is under cognitive control. When a new color memory is expected soon, VWM for color is kept active, leading to interference from the current color memory.

**Fig. 4:**
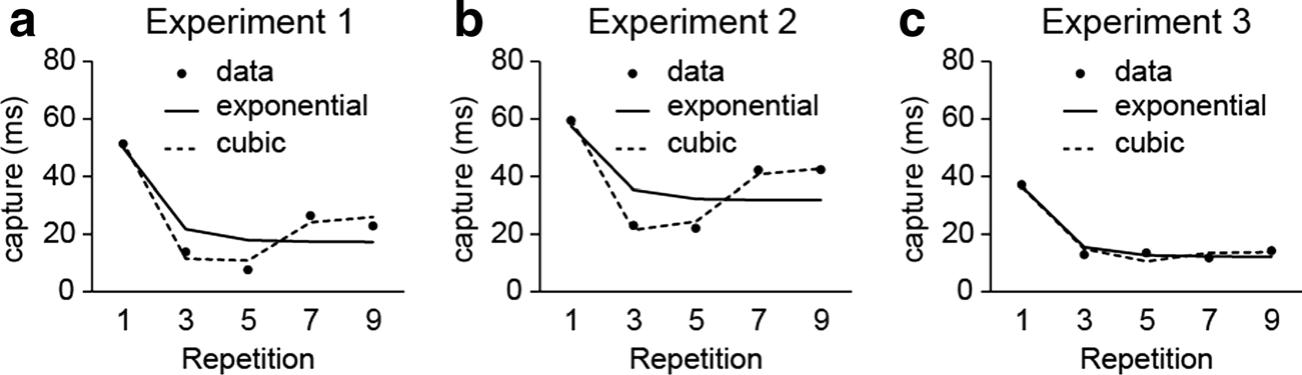
Experiments 1–3: Exponential (solid) and cubic (dashed) regression lines, as fitted to the data (circles) in (a) Experiment 1, (b) Experiment 2, and (c) Experiment 3. The corresponding Akaike information criterion estimates are shown in Table 1.

Surprisingly, as in Experiment 1, in the nine-repetitions blocks, memory-based interference reemerged again at the final repetitions, with the ANOVA on Repetitions 5, 7, and 9 suggesting the same interaction as in Experiment 1 [*F*(2, 34) = 3.074, *p* = *.*059]. To further explore this pattern, we collapsed the data from Experiments 1 and 2 and fitted both monotonic (linear, exponential) and nonmonotonic (quadratic, cubic) regression models. Table 1 and Fig. 4 show that the cubic model provided the best fit to the data, confirming what the data patterns already suggested—namely that memory-based interference across repetitions was characterized by multiple components: an initial decrease, followed by an increase toward the end. Although the focus of our study was on any repetition-related decline in interference, we decided to investigate this rebound effect further in Experiment 3.

**Table 1:** Different model fits per experiment, as expressed by Akaike information criterion values (Akaike, 1998)

	Linear	Exponential	Quadratic	Cubic
Experiments 1 + 2	2,493	2,477	2,477	2,471
Experiment 3	710	704	710	713

## Experiment 3: reactivating VWM is under cognitive control

The finding that the initial offloading from VWM to LTM is under cognitive control may also provide an explanation for the resurgence of memory-based interference. Just as observers decide to keep VWM online when they anticipate an imminent change in color (as in the three-repetitions blocks of Exp. 2), they may decide to bring VWM back online at the end of a nine-repetition sequence, to facilitate the encoding of a new, upcoming memory. A study by Reinhart and Woodman (2014) provided evidence that VWM can be strategically reinstated, even though the object in memory no longer requires VWM. They used the CDA to track VWM’s involvement during repeated search, and found that with repetition of the search target the CDA amplitude decreased, consistent with an offloading of the target representation to LTM. Importantly, when a large reward was then promised, the CDA amplitude returned to starting levels, suggesting that VWM was strategically recruited again in anticipation of the rewarding trial. Interestingly, other experiments using the CDA method have also provided hints of reinstatement of VWM toward the end of repetition sequences, without any promise of reward (Exp. 3 in Carlisle et al., 2011; Exp. 2 in Gunseli, Meeter, & Olivers, 2014).

Although our observers had no obvious incentive to reinstate VWM, in all experiments the length of the sequence was kept constant, and at least a subset of observers might have developed an estimate of when a new color would appear, and resumed VWM-based control in its anticipation. Here we tested whether the strategic reinstatement of VWM indeed accounts for the observed reemergence of memory-based interference. We used the same procedure as in Experiment 1, except that at the end of the sequence, participants first had to perform a *different* task that did not require them to remember a new color. Only after completing this intermediate task did they return to the color memory task. We reasoned that there should now be no reason to reinstate VWM for color toward the end of the repetition sequence, and thus we should not observe a return of memory-based interference. Alternatively, it is possible that no active anticipation was involved, and that the increased interference resulted from, for example, automatic long-term priming mechanisms taking over the guidance of attention as learning accumulated (Kruijne & Meeter, 2015). In that case, we should still observe increased interference at the end of the repetition sequence.

### Method

The method was the same as in Experiment 1A, except for the following changes. Fifteen new volunteers (seven females, eight males; age 19–30 years, *M* = 24 years) participated. Here, after every nine trials, following a 2,000-ms interval, participants switched to an intermediate task. To minimize memory requirements, this task was very similar to the search task; however, no color was involved (i.e., none of the distractor disks was colored). To further ensure that this task was easily dissociated from the regular search task, the stimuli were not presented simultaneously, but appeared sequentially. Every 750 to 1,250 ms, a randomly selected item was added to the display, until all items were visible. Participants had to respond immediately when the diamond appeared by pressing the button that corresponded to the matching search location.

### Results and discussion

The data were analyzed as in Experiment 1. Performance on the intermediate task is reported in the supplementary material.

#### Search RTs

**Fig. 5:**
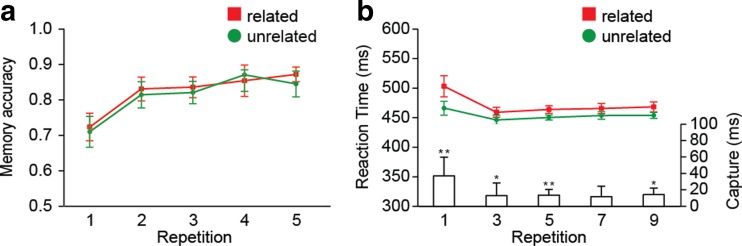
Experiment 3: (**a**) Memory accuracy and (**b**) reaction times as a function of repetition and distractor type

Figure 5 shows the RT results. Exclusion of incorrect search trials (0.5 %) and data trimming (2.3 %) resulted in an overall loss of 2.8 % of the data. We found a main effect of distractor type [*F*(1, 14) = 29.06, *p* < .001, *η*_p_^2^ = .68] and a main effect of repetition [*F*(4, 25) = 15.06, *p* < .001, *η*_p_^2^ = .52]. The interaction approached significance (*F* = 3.00, *p* = *.*074). The pattern of interference in the first part of the sequence replicated that of Experiment 1 and the nine-repetitions blocks of Experiment 2, as interference was reduced from Repetition 1 to Repetitions 3 [*t*(14) = 1.76, *p* = *.*10] and 5 [*t*(14) = 2.51, *p* = *.*03]. However, in contrast to the earlier experiments, now we saw no evidence for an increase in interference toward the end of the sequence (*t*s < 0.31, *p*s > .25). Akaike information criterion estimates (see Table 1) now also clearly favored a monotonic exponential decrease over a higher-order model.

The lack of any resurgence of memory-based interference suggests that it can be suppressed, and is thus under cognitive control. Whereas the previous experiments showed resurgence of memory-based interference prior to the storage of a new color in VWM, the present experiment showed no such increased interference prior to a task for which little working memory was required, and certainly no working memory for color. Taken together, the reemergence of memory-based interference appears to have been driven by the anticipated role that VWM would play in the updating of task-relevant memories.

## General discussion

We investigated how learning representations that are only prospectively relevant (i.e., for a later task) affect visual attention on the current task. Earlier evidence had indicated that such accessory memory items, when represented in VWM, interfere with current selective attention tasks (Olivers et al., 2006; Soto et al., 2005). Here, we assessed whether repeated accessory memories continue to guide attention when they are transferred from VWM to LTM. All experiments demonstrated that the *first* occasion an accessory item was encountered, and thus presumably held in VWM, memory-based interference occurred during search. However, within three repetitions of the accessory memory, interference reached a minimum, indicating that with learning, its effect on attentional selection rapidly diminishes.

Previous research indicated that memory-related capture should be attributed to active maintenance of the memory representation, and not to passive priming effects (Olivers et al., 2006; Soto et al., 2005). The present results support this finding by demonstrating that priming contributes little, even when the accessory memory is presented repeatedly. Whereas memory improved across repetitions, memory-related attentional capture actually decreased. Furthermore, the speed with which active maintenance of the color appeared to be reduced matches similar reductions found in studies using the CDA as a measure of activity (Carlisle et al., 2011; Gunseli, Meeter, & Olivers, 2014; Gunseli, Olivers, & Meeter, 2014).

The finding that memory improves while attentional guidance diminishes is in line with the idea that not the *quality*, but the *status* of a memory representation determines whether or not it will guide attention (Hollingworth & Hwang, 2013; Olivers, Peters, Houtkamp, & Roelfsema, 2011; van Moorselaar, Theeuwes, & Olivers, 2014), and extends this idea to LTM. Where others have assumed *increased* attentional guidance from LTM as search performance improved with target repetition (Carlisle et al., 2011; Gunseli, Meeter, & Olivers, 2014; Gunseli, Olivers, & Meeter, 2014; Reinhart & Woodman, 2014), here we found *decreased* attentional guidance from repeated accessory memories. Taken together, this points to an important functional dissociation within LTM: Observers can learn either to use a currently relevant memory for attention or to shield a prospectively relevant memory from attention.

Also, the results demonstrate that the learning-based attentional status of memory contents is context-dependent, since both the initial reduction and the subsequent reappearance were sensitive to overall task expectations. We believe that these effects should be attributed to the level of VWM involvement during maintenance. As was shown in Experiment 2, even though three repetitions were sufficient to reduce memory-based interference, this reduction was only observed when participants knew the memory would be repeated on subsequent trials. In contrast, when a memory update was expected soon, interference did not diminish, suggesting that the memory was kept active in VWM, and thus guided attention. Similarly, Experiment 3 showed that the increased interference toward the end of the repetition sequence was no longer present when observers switched to an intermediate task that required no color memory whatsoever before starting a new memory sequence. This argues against long-term priming mechanisms driving the increase in memory-based interference observed in Experiments 1 and 2. Instead, we propose that VWM was reinstated in expectation of a new color memory, even though the current memory no longer required VWM. A similar mechanism of reinstating VWM was shown by Reinhart and Woodman (2014). In that study, observers had a clear incentive to reinstate VWM, because it served to supplement the cognitive control already afforded by LTM. In our study, VWM reinstatement, if anything, interfered with search, yet it was not suppressed. This raises the question of whether VWM was strategically reinstated to regain task control, or whether VWM is automatically recruited when anticipating a changing task environment. Future research will be necessary to dissociate these options.

Finally, it is worth pointing out that, even though there was a rapid reduction with repetition, interference from the accessory memory item never completely disappeared (except in Exp. 1A), a finding consistent with earlier studies that have shown some remaining markers of VWM involvement after search target repetition (Carlisle et al., 2011; Gunseli, Meeter, & Olivers, 2014; Gunseli, Olivers, & Meeter, 2014; Reinhart & Woodman, 2014). One could argue that VWM needs to stay involved in order to maintain at least some level of control and flexibility, given the complexity of the task, as well as the fact that the specific memory representations are only relevant within the context of the experiment. Alternatively, it is possible that the handoff from VWM to LTM is actually complete, and that attentional guidance by accessory memories from LTM is simply less strong. Future research will need to dissociate these options.

We conclude that learning prospective memories affects their attentional status, but at the same time increases cognitive flexibility, since different memory systems can be recruited depending on the task context.

## Electronic supplementary material

Below is the link to the electronic supplementary material.

ESM 1 (DOCX 21.5 kb)
