# Supplementary material for: Learning changes the attentional status of prospective memories
Source: Psychon Bull Rev. 2016 Feb 18;23(5):1483–90. doi: 10.3758/s13423-016-1008-7 (PMC5050236; doi:10.3758/s13423-016-1008-7)
Supplement: Supplementary file 1 — (DOCX 21.5 kb) [file 13423_2016_1008_MOESM1_ESM.docx]

**Supplemental Material**

Supplemental analyses for Experiment 1A and 1B separately

*Search RTs*. Separate ANOVA’s on Experiment 1A and 1B yielded main effects of distractor type (*F* (1,14) = 57.18, *p* < 0.001, η*_p_*² = 0.80 in Experiment 1A; *F* (1,14) = 25.93, *p* < 0.001, η*_p_*² = 0.65 in Experiment 1B) and repetition (*F* (4,56) = 20.57, *p* < 0.001, η*_p_*² = 0.60 in Experiment 1A; *F* (1,20) = 10.49, *p* = 0.002, η*_p_*² = 0.43 in Experiment 1B). In both experiments there were significant distractor type by repetition interactions (*F* (3, 36) = 8.75, *p* < 0.001, η*_p_*² = 0.38 in Experiment 1A; *F* (4, 56) = 4.25, *p* = 0.004, η*_p_*² = 0.23 in Experiment 1B). Table 1 shows the different model fits for Experiment 1A and 1B combined and for Experiment 1A and 1B separately.

Table 1: Different model fits per experiment as expressed by Akaike Information Criterion (Akaike, 1998).

|  | Linear | Exponential | Quadratic | Cubic |
| --- | --- | --- | --- | --- |
| Experiment 1A + 1B | 1467 | 1448 | 1448 | 1441 |
| Experiment 1A | 750 | 735 | 735 | 726 |
| Experiment 1B | 723 | 717 | 718 | 719 |
| Experiment 2 | 986 | 984 | 985 | 985 |

*Search accuracy*. Search accuracy was high at 98.5% on average in Experiment 1A and 98.7% on average in Experiment 1B. The analysis on search accuracy yielded a main effect of distractor type *F* (1, 28) = 12.96, *p* = 0.001, η*_p_*² = 0.32), a significant experiment by distractor type interaction *F* (1, 28) = 10.20, *p* = 0.003, η*_p_*² = 0.27) and a significant three-way interaction *F* (4, 112) = 3.28, *p* = 0.014, η*_p_*² = 0.11). All other tests were non-significant (all *F*’s < 1.43, all *p*’s > 0.230). Separate ANOVA’s showed a main effect of distractor type in Experiment 1B (*F* (1,14) = 30.77, *p* < 0.001, η*_p_*² = 0.69), which was driven by better performance in the unrelated condition. All other tests were non-significant (all *F*’s < 1.83, all *p*’s > 0.136), although the distractor type by repetition interaction was close to significance in Experiment 1A (*F* = 2.76, *p* = 0.077). This pattern followed that of the RTs.

*Memory accuracy*. The repeated-measures ANOVA of the memory scores produced a main effect of repetition (*F* (4, 112) = 56.34, *p* < 0.001, η*_p_*² = 0.67) and a significant experiment by repetition interaction (*F* (4, 112) = 3.38, *p* = 0.012, η*_p_*² = 0.11). All other tests were non-significant (*F*’s < 2.80, *p*’s > 0.11). Separate ANOVA’s yielded main effects of repetition in both Experiment 1A (*F* (4,56) = 42.65, *p*< 0.001, η*_p_*² = 0.75) and 1B (*F* (4,56) = 20.44, *p*< 0.001, η*_p_*² = 0.59), reflecting increasing accuracy on the memory test with repetition of the memory item. There were no other effects (all *F*’s < 2.90, all *p*’s > 0.111).

Supplemental analyses for Experiment 2

*Search accuracy*. Search accuracy was high at 97.5% on average in the nine repetitions block and 98.0% on average in the three repetitions block. In the nine repetitions block, analysis on search accuracy showed no effects (all *F*’s < 1.81, all *p*’s > 0.136; although the main effect of distractor type, reflecting better performance in the unrelated condition, approached significance (*F* = 4.21, *p* = 0.056). In the three repetitions block there were no effects (all *F*’s < 1.15, all *p*’s > 0.250). Model fits are shown in Table 1.

*Memory accuracy*. The repeated-measures ANOVA on the memory scores produced a main effect of repetition in the nine repetitions (*F* (4, 68) = 25.87, *p* < 0.001, η*_p_*² = 0.60) and the three repetitions block (*F* (1, 17) = 38.64, *p* < 0.001, η*_p_*² = 0.69), both reflecting better memory performance with repetition. In the three repetitions block there was a main effect of distractor type (*F* (1,17) = 7.06, *p* = 0.017, η*_p_*² = 0.29) showing better memory performance in the related than in the unrelated condition. No other effects were significant (all *F’*s *<* 3.21, all *p’*s > 0.091).

Supplemental analyses for Experiment 3

*Search accuracy*. Search accuracy was high at 99.5% on average. The analysis on search accuracy showed no effects (all *F*’s < 0.60, all *p*’s > 0.25).

*Memory accuracy*. The repeated-measures ANOVA on the memory scores, produced a main effect of repetition (*F* (4,56) = 15.88, *p* < 0.001, η*_p_*² = 0.53) reflecting better memory performance with repetition. No other effects were significant (*Fs <* 1.23, *p* > 0.250).

*Intermediate task.* Intermediate task performance was high with a mean accuracy of 96.6% correct and a mean RT of 508 ms.
